# Naturalistic multimodal emotion data with deep learning can advance the theoretical understanding of emotion

**DOI:** 10.1007/s00426-024-02068-y

**Published:** 2024-12-21

**Authors:** Thanakorn Angkasirisan

**Affiliations:** Department of Experimental Psychology, University of Oxford, Oxford, UK

## Abstract

What are emotions? Despite being a century-old question, emotion scientists have yet to agree on what emotions exactly are. Emotions are diversely conceptualised as innate responses (*evolutionary view*), mental constructs (*constructivist view*), cognitive evaluations (*appraisal view*), or self-organising states (*dynamical systems view*). This enduring fragmentation likely stems from the limitations of traditional research methods, which often adopt narrow methodological approaches. Methods from artificial intelligence (AI), particularly those leveraging big data and deep learning, offer promising approaches for overcoming these limitations. By integrating data from multimodal markers of emotion, including subjective experiences, contextual factors, brain-bodily physiological signals and expressive behaviours, deep learning algorithms can uncover and map their complex relationships within multidimensional spaces. This multimodal emotion framework has the potential to provide novel, nuanced insights into long-standing questions, such as whether emotion categories are innate or learned and whether emotions exhibit coherence or degeneracy, thereby refining emotion theories. Significant challenges remain, particularly in obtaining comprehensive naturalistic multimodal emotion data, highlighting the need for advances in synchronous measurement of naturalistic multimodal emotion.

## Introduction

One of the earliest scientific studies on emotions dated back to Darwin’s (1872) *The Expression of the Emotions in Man and Animals*. He observed that human and nonhuman animals reliably display or express facial and body movements in similarly recognisable patterns, so he theorised, following assumptions from his theory of evolution by natural selection, that these expressive movements are a product of selective pressures over generations which reflect the state of mind or emotions to serve adaptive functions (Darwin, 1872). This laid the foundation for future scientific studies on emotions grounded in empirical observation and formed a basis for the evolutionary view of emotions. Since Darwin’s time, several theories of emotions have been developed and tested, yet the field still has little consensus about what an emotion really is.

In the first part of this review, the author provides an overview of the contemporary views on emotions (i.e., evolutionary, constructivist, appraisal, and dynamic systems), focusing on their theoretical assumptions and empirical supports. In light of the emerging artificial intelligence (AI) revolution, the second part of this review argues for a data-driven approach utilising multimodal emotion data in the wild and deep learning algorithms as research tools that might unify or refine existing understanding of emotions. The last part provides examples of how future research could use these AI tools to analyse multimodal emotion datasets to expand our theoretical understanding of emotion.

## Perspectives on emotions

### Evolutionary view

At the cornerstone of emotion science lies the evolutionary, or classical, view of emotions, prominently represented by basic emotions theory (BET). According to BET, selective pressures from our evolutionary past favour a set of natural, discrete, primary emotions—happiness, sadness, surprise, disgust, fear, and anger—which are universally hardwired to enable individuals to adaptively respond to fundamental life tasks (Ekman, 1992). Each basic emotion has unique elicitors (Lench et al., 2011), physiological signatures (Izard, 2007; Panksepp, 1998), and expressive behaviours that can be recognised primarily through facial muscle movements and are minimally affected by cultural context (Ekman, 1992). The support for the universality of basic emotions came from the study where Paul Ekman travelled to New Guinea and other regions of the world with photos of facial expressions representing the six emotions. This is to investigate whether people from various cultures, even those from culturally isolated tribes, could recognise the six discrete emotions from the photos. He found that emotion recognition accuracy was consistently high and above chance levels for all six emotions across cultures, aligning with the assumption of the universality hypothesis and BET (Ekman & Friesen, 1971; Ekman et al., 1969). Over decades, this result has been replicated over a hundred times (Elfenbein & Ambady, 2002), making BET an empirically grounded theory that has remained a central narrative in emotion science for over half a century.

The evolutionary view of emotion, nevertheless, has not stood without criticism. Relatively newer studies that employed more sophisticated and robust methods concluded that there was an inconsistency with BET’s assumptions (Duran & Fernandez-Dols, 2021). For instance, a cross-cultural study found that Himba people of Namibia who had little exposure to Western culture, when asked to sort emotional expressions in faces into the same emotion categories without emotion label from the experimenter, did not sort facial expressions into the same emotion categories as US people (Gendron et al., 2014). A comprehensive meta-analysis of hundreds of neuroimaging studies of emotions did not find dedicated neural circuits for distinct emotions (Lindquist et al., 2012). Although BET supporters defended the classical view by highlighting the flaws and alternative interpretations in those studies (e.g., Adolphs, 2017; Ekman, 1994; Keltner et al., 2019; Panksepp, 2007), other groups of emotion scientists resorted to alternative views.

### Constructivist view

The psychological constructivist view claims that emotions are not hardwired but are constructed by the mind from the combination of more basic psychological and neural processes (Barrett, 2017; Russell, 2009). Built on the two-dimensional model of affect, which suggests that all emotions are reducible to a two-dimensional affective state, namely valence and arousal (i.e., Russell, 1980), the theory of constructed emotion (TCE) posits that people construct emotional feelings (subjective experience) and meaning (emotion concept categories) by integrating interoception of affective dimensions and interpretation of contextual information through learning (Barrett, 2017). For instance, if an individual’s brain anticipates encountering a bear and the associated negative feelings that might arise from a bear attack, it might construct and categorise the experience as fear. Constructivist theories offer an alternative explanation for the lack of localised brain basis of discrete emotion categories (Lindquist et al., 2012) and increasing evidence for the role of context and language in emotion perception (Barrett et al., 2011; Lindquist & Gendron, 2013). Despite that, our poor understanding of how brain computations create different relevant psychological phenomena has limited our ability to examine how the brain directly constructs emotions. The supporting evidence for the constructivist perspective, thus, relied on studies on emotion semantics that vary between language and culture (Jackson et al., 2019; Lindquist, 2017).

### Appraisal view

Beyond the evolutionary and constructed theories, which are complete rival perspectives on emotions, the appraisal theories have both biological and non-biological versions. The key uniqueness of the appraisal view is that it emphasises that emotions depend on how individuals interpret or appraise situations (Frijda, 1986; Lazarus, 1991; Scherer, 2009; Smith & Ellsworth, 1985). According to this view, emotions are not direct reactions to events themselves but rather to the cognitive appraisal of those events along dimensions such as goal congruence, expectedness, certainty, agency, and controllability (Moors et al., 2013; Scherer, 2009; Smith & Ellsworth, 1985). This explains why similar situations can lead to different emotions across individuals and even within the same person at different times. For instance, in a traffic jam, one may feel angry if they perceive the delay as a severe disruption to their goal of arriving on time, thus perceiving it as an obstacle. Another person, not in a rush, might appraise the situation as a minor inconvenience, eliciting no strong emotional response.

Empirical support for appraisal theory comes from studies that assess how specific cognitive evaluations correspond to distinct emotions. One study examined cognitive appraisals of stressors, such as a college exam, to determine their influence on emotional responses and coping strategies. Results indicated that students viewing a college exam as a threat tended to feel anxious, whereas those seeing it as a challenge felt confident, underscoring the impact of appraisals on emotional experience (Folkman & Lazarus, 1985). Similarly, distinct patterns of cognitive appraisal, like goal relevance, fairness, and control, could be linked to different emotions, such as anger, sadness, and happiness, further reinforcing the idea that emotions emerge from cognitive evaluations along various dimensions (Smith & Ellsworth, 1985). While appraisal theory has faced critiques of its own (see Moors, 2010, 2013, 2022), it provides valuable insights into how emotions are shaped by subjective interpretations and fits into broader understanding that cognition is an integral part of emotion.

### Dynamical systems view

The dynamical systems approach offers a metaphoric framework for understanding emotions. In this view, emotions are conceptualised as emergent, self-organising, adaptive responses that emerge through interactions among various interconnected systems, including stimuli, cognitive processes, physiology, expressions, subjective experiences, and emotion concepts (Lewis, 2005; Wood & Coan, 2023). Central to this framework is the concept of *attractor-like states*—stable self-organising patterns of subjective experience, physiology, and expression in a multidimensional state space that manifests as recognisable emotions like joy, anger, or sadness. Over time, the configuration patterns that reliably lead to successful goal-directed pursuit are reinforced and become stable *attractor-like states* (Wood & Coan, 2023). Multiple patterns of neural and physiological activity produce the same stable emotional state and functional outcome, with these patterns being only probabilistically associated with specific emotions rather than having a one-to-one mapping (Doyle et al., 2022; Hoemann et al., 2020; Wood & Coan, 2023). This emotional landscape gradually stabilises throughout childhood and adolescence from experience (Reitsema et al., 2022). While emotions vary across individuals and cultures, common patterns emerge due to shared ecological pressures and biological systems (Lindquist et al., 2022; Wood & Coan, 2023). This dynamical systems perspective provides nuanced insights that complement existing evolutionary and constructivist theories, though it requires further empirical validation.

## Unique methodological focus, unique perspective

Each emotion perspective has its own strengths and weaknesses, which have been extensively reviewed in previous works (Moors, 2022). Notably, supporting evidence for each view was derived from different methodological approaches influenced by their pre-assumptions (Fox, 2018). For instance, those taking the constructivist perspective referred to evidence primarily from semantic analysis of self-reported subjective experience, which is context-sensitive. By contrast, those taking the evolutionary view drew evidence from one-to-one mapping between a pre-defined set of emotion categories and their neurophysiological or expressive reactions. Appraisal theorists seek to map evaluative patterns of stimuli to distinct emotional experiences. This limited range of methodologies used to test each emotion theory can only obtain empirical evidence that either confirms or contradicts a specific assumption of the theory it was specifically designed to test, leading to fragmented empirical literature on emotion theories. To move toward a consensus on the nature of emotions, it is essential to develop methodological approaches capable of testing multiple theoretical views and assumptions simultaneously.

## Artificial intelligence enters emotion research

Advancements in data science have brought a new, promising framework to answer the fundamental question about the nature of emotions. For instance, one study utilised big data from the internet by gathering thousands of short video clips that depicted a diverse range of emotional situations and asked nearly a thousand people to either (i) freely label their emotions, (ii) rate their degree of feeling on various emotion categories, or (iii) rate the affective dimensions in response to each video, and pioneered analytic approach from deep learning to map self-reported emotions on a multidimensional semantic space, in which 27 distinct emotion categories were captured bridging by continuous gradients from people’s self-reported experiences (Cowen & Keltner, 2017). This approach provides a richer spectrum of emotion concepts, which appear to be high-dimensional, categorical, and distributed in gradients, that could not be captured from traditional emotion research that relied on limited data from only facial expressions and one-to-one mapping with narrow six categories of emotion (Cowen & Keltner, 2021). Consequently, similar large-scale statistical modelling and machine learning methods have been applied to study people’s recognition of emotions from expressive behaviours and identify brain correlates of high-dimensional emotions, revealing similar patterns. Specifically, brief vocalisation and facial-bodily expression were found to convey at least 24 distinct emotion categories distributed in gradients (Cowen & Keltner, 2020; Cowen et al., 2019), and fMRI patterns in multiple brain regions can accurately predict dozens of emotions (Horikawa et al., 2020).

Such AI-driven techniques have also been extended to study emotions across cultural contexts. One study trained deep neural networks on multidimensional emotion ratings of 186,744 facially expressive YouTube clips and used the trained model to analyse the extent to which facial expressions occur in similar real-world contexts in six million videos across 144 nations and found that 16 facial expressions can be reliably and differentially associated with specific contexts like weddings, sports competitions, and fireworks worldwide (Cowen et al., 2021). Similarly, people in different cultures reliably attribute facial expressions and vocal bursts to distinct emotional meanings (Brooks et al., 2023, 2024; Cowen et al., 2024; Laukka & Elfenbein, 2021). These findings form a basis that, although some cultural variations exist, the way people in the modern world express emotions can be reliably predicted, suggesting similarity across cultures.

Findings from AI-driven studies that map emotion labels across various modalities onto a multidimensional space suggest nuanced properties about the dimension, distribution, and conceptualisation of emotions (Cowen & Keltner, 2021). First, data across different modalities show that emotions are multifaceted, existing within a space defined by multiple axes, with at least two dozen distinct dimensions. This number of dimensions far exceeds BET’s six emotion categories and TCE’s valence and arousal dimensions. However, this does not contradict the assumption from either appraisal or dynamical systems perspective. Second, emotions are arranged in gradients within the semantic space, with overlapping patterns and less defined boundaries between related categories, most aligning with the assumption from constructivist and dynamical systems views. Third, distinct emotion categories across modalities appear to provide the best explanatory power, most consistent with the explanations from both evolutionary and dynamical systems views. Together, this AI-driven approach, viewing emotions as conceptually and computationally multifaceted, demonstrates that traditional theories, while valuable, remain incomplete, likely due to their narrow focus on specific aspects of multi-componential emotions.

The findings from AI-driven studies, nevertheless, also warrant careful interpretation of its contribution to the fundamental understanding of emotions. So far, our example studies rely on supervised learning models that learn to recognise patterns corresponding to the preassigned labels. However, some evidence suggests that the patterns identified by supervised models often differ from those captured by unsupervised models, which uncover structures without relying on preassigned labels (Azari et al., 2020). This discrepancy raises a question about whether supervised models can truly capture objective emotion categories or merely reflect subjectively meaningful taxonomies of emotions.

Still, these AI-driven studies demonstrate the transformative potential of AI-driven approaches, particularly deep learning, as research tools, allowing researchers to leverage much larger, more naturalistic, and more complex datasets, moving beyond the constraints of traditional research paradigms to give us deeper insights into the complex nature of emotions. For example, evolutionary theorists have long claimed that distinct emotion categories are linked to unique physiological, experiential, and expressive responses (Ekman, 1992). However, studies that examined multimodal emotion responses have led to inconsistent conclusions about the speculated coherence nature of emotions (Mauss et al., 2005). Generally, research showed high coherence between experiential and expressive emotion systems but lower coherence with physiological emotion systems (Mauss et al., 2005). Top-down processes like emotion regulation and body awareness were found to moderate the coherence between emotional experiences and physiological responses (Dan-Glauser & Gross, 2013; Sze et al., 2010). In the traditional sense, high coherence between emotional experiences and expressions is aligned with the evolutionary view, whereas the evidence for top-down influences on emotional experience and physiology coherence is more consistent with the constructivist view. This variability in emotion coherence across modalities also hints at the possibility of emotions being emergent, self-organising states, as suggested by the dynamical systems view. The emotion coherence problem could be approached by integrating data from multimodal markers of emotions along with contextual factors in deep neural network models that learn to capture patterns of associations among contexts, emotional experiences, expressions, and brain and bodily responses and map them along a multidimensional space. This descriptive exploration of the intricate relationships between multimodal emotion systems would provide revolutionising insights that allow simultaneous testing of multiple theories of emotions or shed light on new integrated theories.

## Multimodal markers of emotions

To achieve a comprehensive, integrative model of emotion that tests multiple theories at once, massive datasets from various modalities of emotions, preferably in naturalistic settings, have to be gathered. Deep learning algorithms will come to be useful for such massive and complex datasets (Lin et al., 2023). While the technical details and implementation of these algorithms have been extensively discussed elsewhere (see Urban & Gates, 2021; Zhang & Tan, 2024), this article focuses on their conceptual applications, leaving model-specific intricacies aside due to space constraints. The multimodal emotion data can be conceptually organised into four interconnected systems: subjective experience, contextual factors, physiological responses, and expression behaviours. These systems interact dynamically, providing a rich, multidimensional view of emotional phenomena.

The first system is the psychological or experiential system of emotions. The commonly studied modality within this system is the subjective experience of emotion, often measured by self-report. Emerging technologies allow momentarily sampling of self-reported emotions using smartphone prompts, providing insights into subjective emotional experiences in dynamic real-world contexts (Hoemann et al., 2023). However, the subjective nature of this experiential modality means that major contextual factors like language and culture, as well as cognitive processes like appraisal and emotion regulation, can influence emotional experiences and, therefore, should be accounted for. Language and culture can be integrated into models through representative data and appropriate labelling. By contrast, appraisal and emotion regulation are harder to systematically measure in dynamic, naturalistic contexts. These processes are shaped by highly individualised factors such as goals and values, making them challenging to quantify. Addressing these factors is essential to avoid oversimplifying emotional experiences and accurately understanding their interactions with other systems, such as physiological responses (Porges et al., 1994).

The second system is contextual factors, encompassing the intricate environmental, cognitive, and cultural influences that shape emotional perception, dynamics, and responses. This system examines how individuals extract goal-relevant features from their surroundings to adaptively respond to emotional stimuli. Contextual factors operate at multiple levels—external, such as environmental stimuli and sociality, and internal, such as culture, values, goals, and cognitive styles—and interact in complex, often unpredictable ways. These interactions make it exceedingly difficult to comprehensively account for the full scope of contextual influences on emotions. Researchers have begun leveraging advanced computational methods to measure and analyse contextual factors. Convolutional neural networks can process visual environmental data to identify features—such as object, race, and gender—that might affect how agents emotionally react. Wearable sensors, including microphones, can capture auditory cues like vocal tone and prosody, while natural language processing tools can analyse text data to infer conversational influences. Furthermore, virtual reality offers an experimental platform to precisely manipulate contextual variables, enabling controlled studies of their effects on emotional perception and behaviour (Kako et al., 2023). These tools represent significant advancements, yet integrating such complex contextual factors into coherent models of emotion remains a formidable challenge to overcome.

The third system is the physiological modalities of emotion. Numerous physiological modalities of emotions have been studied in the research, from markers in the central nervous system, namely brain activity (Lindquist et al., 2012; Panksepp, 1998), to the autonomic nervous system, such as heart rate, respiration, skin temperature, and electrodermal activity (Mauss & Robinson, 2009; Saganowski et al., 2022). These physiological responses are traditionally measured by placing a sensory device near the body or relevant organ to detect bodily signals. Although there are advances in measuring bodily signals using wearable biosensors (e.g., smartwatches) making it possible to track the naturalistic physiological activities of an individual on a day-to-day basis (Hoemann et al., 2020), some physiological modalities (e.g., neural activity) are more complicated to do so at large-scale, posing another challenge to gather rich naturalistic data from the emotion physiology systems for AI-driven research. Overcoming these challenges requires interdisciplinary innovations like a wearable brain-bodily tracking device.

The fourth system is expressive behaviours, including but not limited to facial expressions, body postures, and vocal bursts. The emotion expression system is easy to observe and measure in naturalistic contexts. Data on emotional expressions have been extensively studied from publicly available emotion-rich pictures, videos, and audio online (e.g., Cowen et al., 2021). Emerging technologies, including devices like Apple Vision Pro and Meta’s Orion Augmented Reality glasses, hold great promise for research on multimodal emotion integration by enabling researchers to synchronously record the dynamics of facial or vocal expressions as people engage with real-world environments. These multimodal recording tools offer unprecedented opportunities for studying the temporal dynamics of expressive behaviour and its coordination with other emotional systems. For instance, researchers can analyse how changes in vocal pitch correspond to shifts in facial expressions during high-stress situations, providing deeper insights into emotional synchrony across modalities.

## Emotion (data) science and directions forward

In the age of AI, better quality and larger quantity of data can provide deeper nuance, making it crucial for emotion science to obtain massive naturalistic datasets across all modal markers of emotions. However, as highlighted by current limitations in emotion measurement, obtaining such all-encompassing datasets remains a significant challenge. For now, researchers can leverage existing multimodal datasets that focus on non-brain physiological responses, facial expressions, subjective experiences, and contextual information (e.g., Chen et al., 2021; Saganowski et al., 2022). These more accessible modalities offer a practical starting point for exploring the foundational principles of emotions while addressing questions about how coherence or fragmentation manifests across different modalities.

One promising direction is to explore how supervised learning models can reliably capture the pattern of associations between expressions, physiological responses, contexts, and self-report experiences of emotions in multidimensional spaces. Past works have demonstrated the ability of supervised learning models to map one specific emotion modality to another specific emotion modality, particularly mapping contexts to emotional experiences (Cowen & Keltner, 2017), expressive behaviours to emotional experiences (Brooks et al., 2023, 2024; Cowen & Keltner, 2020; Cowen et al., 2019, 2024), contexts to expressive behaviours (Cowen et al., 2021), and neurophysiological activity to emotional experiences (Horikawa et al., 2020). How the configuration of patterns across multimodal markers of emotion maps onto a multidimensional space of a specific modality could reveal more nuance to the associations across interrelated emotion systems. For instance, patterns of expression, physiology, and contexts might not only more reliably predict emotional experience than the pattern of physiological responses alone, but they might also reveal interactions and intricacies, reflecting the deeper underlying structure of emotional phenomena within the interactive systems beyond the association being captured from mapping one marker of emotion to the other.

Despite the usefulness of emotion taxonomy derived from supervised learning models serving as a data-driven framework for classifying and predicting emotion with great real world application (e.g., in human-computer interaction), such a method can only provide evidence for emotion coherence across multimodal markers of emotion. Comparing such supervised multimodal emotion models with unsupervised models is important to gain insights into whether such mapping naturally emerged or is virtually guided by our concept of emotions. Although some research has shed light on the inconsistent pattern clusters captured between supervised and unsupervised models on each modal marker of emotions separately (Azari et al., 2020), the integration of multimodal data adds layers of complexity to the underlying data structure that deep learning models can learn, needing its own comparison. For instance, applying clustering algorithms or self-organising maps to datasets combining expressions, physiological responses, subjective experiences, and contexts could uncover emergent patterns of distinct emotional phenomena. How well the configuration of multimodal emotion patterns emerges into clusters captured from an unsupervised model that fit with the supervised model’s mapping can provide insights into whether recognisable emotion patterns of distinct emotion categories emerge naturally or subjectively.

Moreover, because deep learning models are not deterministic, they learn to give probabilistically best guess estimate of a pattern of emotional responses from the pattern of emotional responses that most commonly co-occur in the data (e.g., people often smile when they feel happy), another crucial line of research involves understanding whether this probabilistic mapping of multimodal emotional responses can be better described by coherence or degeneracy. In other words, are the relationships between different emotion systems best characterised by one-to-one mapping, or are there multiple emotional response patterns that map to the same emotion? Studies could track emotional responses within individuals across diverse scenarios to explore within-person emotion consistency and degeneracy while also examining how generalisable these patterns are across populations and cultures. Such insights would directly weigh on the emotion debate of whether emotion coherence is a reliable feature of emotional systems or not.

Unveiling the complexity of contexts that shape emotions presents a compelling avenue for future research. According to appraisal theories, emotions arise not only from external stimuli but also from individuals’ evaluations of events based on personal goals and values. However, AI-driven emotion research has predominantly focused on the role of external contexts, such as environmental cues or situational dynamics (Cowen et al., 2021), potentially oversimplifying the nuanced interaction between context and emotional experience. Developing computational models that mirror how individuals process environmental stimuli and evaluate them along appraisal dimensions could address this gap. Such models should aim to capture the distinct patterns of multimodal emotional responses associated with specific appraisal profiles, including factors like perceived control, relevance, or congruence with personal goals. For instance, agent-based approaches, including reinforcement learning, could simulate the dynamic interactions between internal contexts—like goals, past experiences, or values—and external environments over time, offering a framework to explore how environmental cues are appraised and shape the complexity of emotional processes.

These proposed research directions aim to address pressing questions in emotion science. While these suggestions are not exhaustive, they highlight the potential for multimodal approaches to inspire future research in the field. By tackling the coherence problem and other open questions with techniques from data science, emotion science can move closer to understanding the complexity of emotional systems in their full richness.

## Conclusion

In recent years, the application of AI-driven methods, relying on deep learning models of big emotion data, in the scientific study of emotions promises to move beyond unique perspectives on emotions that were traditionally derived from different methodological paradigms. The future of emotion research lies in harnessing AI tools to explore the relationships across multimodal markers of emotions. Integrating physiological, experiential, contextual and expressive data within multidimensional spaces offers an integrative model of emotions that could yield deeper insights into the coherence and diversity of emotional phenomena. Advances in computational power, data collection technologies (e.g., multimodal wearables), and interdisciplinary collaborations are starting to make such projects more realistic. The potential for a comprehensive, integrative model of emotion that tests multiple theories at once is an exciting prospect. This approach may soon become a viable and transformative area of research in emotion science.

## Data Availability

No datasets were generated or analysed during the current study.
